# Physical Activity Associated Proteomics of Skeletal Muscle: Being Physically Active in Daily Life May Protect Skeletal Muscle From Aging

**DOI:** 10.3389/fphys.2019.00312

**Published:** 2019-03-26

**Authors:** Ceereena Ubaida-Mohien, Marta Gonzalez-Freire, Alexey Lyashkov, Ruin Moaddel, Chee W. Chia, Eleanor M. Simonsick, Ranjan Sen, Luigi Ferrucci

**Affiliations:** Intramural Research Program, National Institute on Aging – National Institutes of Health, Baltimore, MD, United States

**Keywords:** physical activity, proteomics, skeletal muscle, mitochondria, spliceosome, immunity, DNA damage, mass spectrometry

## Abstract

Muscle strength declines with aging and increasing physical activity is the only intervention known to attenuate this decline. In order to adequately investigate both preventive and therapeutic interventions against sarcopenia, a better understanding of the biological changes that are induced by physical activity in skeletal muscle is required. To determine the effect of physical activity on the skeletal muscle proteome, we utilized liquid-chromatography mass spectrometry to obtain quantitative proteomics data on human skeletal muscle biopsies from 60 well-characterized healthy individuals (20–87 years) who reported heterogeneous levels of physical activity (not active, active, moderately active, and highly active). Over 4,000 proteins were quantified, and higher self-reported physical activity was associated with substantial overrepresentation of proteins associated with mitochondria, TCA cycle, structural and contractile muscle, and genome maintenance. Conversely, proteins related to the spliceosome, transcription regulation, immune function, and apoptosis, DNA damage, and senescence were underrepresented with higher self-reported activity. These differences in observed protein expression were related to different levels of physical activity in daily life and not intense competitive exercise. In most instances, differences in protein levels were directly opposite to those reported in the literature observed with aging. These data suggest that being physically active in daily life has strong and biologically detectable beneficial effects on muscle.

## Introduction

The decline in muscle strength is one of the most striking phenotypes of aging, which is only partially accounted for by a reduction in muscle mass, suggesting a loss of cellular and molecular integrity of muscle tissue, and/or impairment of neuromuscular control with aging. Low muscle strength is a powerful, independent predictor of slow gait, mobility disability, and early mortality (Metter et al., 2002; Hicks et al., 2012; Moore et al., 2014). No interventions are currently available that can prevent or attenuate the decline in muscle strength with aging except exercise, especially resistance training. In spite of this evidence, the percentage of people who regularly exercise is still low and this percentage declines with aging (Sallis, 2000; Milanovic et al., 2013). It has been suggested that people who have an active lifestyle in daily life have a slower decline of muscle mass and strength with aging (Paterson et al., 2007; Cartee et al., 2016). Understanding how physical activity in daily life affects muscle physiology in older persons might help in developing new interventions that, by targeting the same mechanisms triggered by physical activity, could prevent the development of muscle impairment with aging. Numerous studies have investigated the impact of a sedentary lifestyle and low physical activity on health outcomes in both younger and older individuals. Physical inactivity, either long or short-term, negatively affects muscle performance and is associated with diminished aerobic capacity, as well as reduced insulin sensitivity and basal metabolic rate (Biolo et al., 2005; Hamburg et al., 2007; Bogdanis, 2012). Furthermore, physical activity alone has been shown to improve and regulate metabolic homeostasis and metabolic efficiency (Palmnas et al., 2018). Overall, an active lifestyle could be conceptualized as a mixture of aerobic and resistance exercise, but the intermittent, and variable mixture of these activities make it difficult to study (Neufer et al., 2015). Endurance and resistance training elicit both common and specific metabolic/morphologic adaptations in muscle, some of which are common between tissues (Wilkinson et al., 2008; Kazior et al., 2016). In general, the stress that is induced by exercise challenges energy homeostasis in myocytes, shifting the cellular environment towards an oxidative state (Egan and Zierath, 2013). This induces microdamage that stimulates both transcriptional and posttranscriptional responses, which then promotes synthesis of specific proteins that seek to reestablish a different homeostatic equilibrium. Endurance training maximally stimulates mitochondrial biogenesis, enhances aerobic metabolism and fatty acid utilization, and produces change in muscle fiber composition (Kohn et al., 2011; Groennebaek and Vissing, 2017). In contrast, heavy resistance training stimulates the synthesis of contractile proteins, leading to muscle hypertrophy, and increases in maximal contractile force speed and output. Whether an active lifestyle is sufficient to activate the same biological mechanisms triggered by endurance and resistance training is unknown. In 2014, an NIH workshop recommended that -omics be used for “Understanding the Cellular and Molecular Mechanisms of Physical Activity-Induced Health Benefits” (Neufer et al., 2015). Indeed, skeletal muscle adaptation to an active lifestyle should be reflected in specific gene expression and, ultimately, proteomic profiles (Izquierdo et al., 2004; Mahoney et al., 2005; Larina et al., 2014).

In recent years, a handful of studies have examined the protein composition of human muscle cell types and tissues (Hojlund et al., 2008; Gonzalez-Freire et al., 2017) including proteomic differences between old and young muscle (Doran et al., 2009; Gueugneau et al., 2014; Lourenco dos Santos et al., 2015; Murgia et al., 2017), athletes and non-athletes (Abe et al., 2018), exercise in extreme conditions (Larina et al., 2014; Schild et al., 2015), and physical activity and metabolic disorders (Goodpaster et al., 2001; Camera et al., 2017; Kleinert et al., 2018). These studies have helped to characterize the physiological adaptations of healthy human muscle to different types of exercise (Rockl et al., 2007; Trappe et al., 2016). Most of these studies focused on the acute and immediate effects of short bouts of high intensity exercise in either human or mice/rat models (Guelfi et al., 2006; Egan and Zierath, 2013; Petriz et al., 2017), as well as long-term effects of exercise (Crane et al., 2013; Mosole et al., 2014; Belaya et al., 2018). However, very little research has focused on assessing the association of daily physical activity with the muscle proteome in healthy community-dwelling individuals. To verify whether an active lifestyle is associated with detectable changes in skeletal muscle and to start to characterize these changes, we performed a quantitative, mass spectrometry-based proteome analysis of muscle specimens from a group of well-characterized healthy individuals with a wide age-range (20–87 years) and who self-reported different levels of physical activity. Independent of age and technical covariates, we found that high levels of physical activity (versus low levels) were associated with an overrepresentation of mitochondrial proteins, tricarboxylic acid (TCA) cycle enzymes, chaperone proteins, and proteins associated with genome maintenance. In contrast, proteins related to the spliceosome and transcription regulation, immune proteins, apoptosis proteins, DNA damage proteins, and senescent proteins were underrepresented in muscle of participants who reported higher physical activity. Differences observed were mostly opposite to those observed with skeletal muscle aging. Interestingly, only some of these differences contradict observations in the literature that have been associated with aging in skeletal muscle.

## Materials and Methods

### Study Design and Participants

Muscle biopsies analyzed in this study were collected from participants from the genetic and epigenetic study of aging and laboratory testing (GESTALT). Potential participants were enrolled in GESTALT if they met the IDEAL (Insight into the Determination of Exceptional Aging and Longevity) criteria; namely if the participants were free of major diseases, except for controlled hypertension or a history of cancer that had been clinically silent for at least 10 years, were not chronically medicated (except one on antihypertensive medication), had no physical or cognitive impairments, had a BMI less than 30 kg/m^2^, and were not professional athletes. Details regarding IDEAL criteria are reported elsewhere and includes information on medical history, physical exams, and blood tests results that were collected and interpreted by a trained nurse practitioner (Schrack et al., 2014). Participants were admitted to 3 days of testing at the Clinical Research Unit of the National Institute on Aging Intramural Research Program. Overall, data and muscle specimens from 60 participants were available for this study. However, two participants were excluded because the muscle specimen provided was too small to obtain reliable proteomic data. Therefore, data from 58 participants dispersed over a wide age-range (20–34 years, *n* = 13; 35–49 years, *n* = 11; 50–64 years, *n* = 12; 65–79 years, *n* = 12; 80+ years, *n* = 10) were used for this study. Participants’ height and weight were objectively assessed. The GESTALT protocol was approved by the Intramural Research Program of the US National Institute on Aging and the Institutional Review Board of the National Institute of Environmental Health Sciences. All participants provided written, informed consent at every visit.

### Muscle Biopsies

Prior to skeletal muscle biopsies, the depth of the subcutaneous fat (uncompressed and compressed) was determined using MRI images of the middle thigh performed on the previous day. A region above the vastus lateralis muscle was marked at the mid-point of a line drawn between the great trochanter and the mid-patella upper margin. The skin was prepped with povidone – iodine (Betadine^®^) and ethyl alcohol, and the outside areas covered with sterile drapes. The biopsy site was anesthetized, first intradermally using a 27-gauge needle and then subcutaneously using a 23-gauge × 1 1/2 -inch needle, follow by an 18-gauge spinal needle, with ∼15 mL of 1% lidocaine in sodium bicarbonate. This method ensured that the subcutaneous tissue and muscle fascia, but not the muscle fibers, were infiltrated with the anesthetic so as not to distort the tissue structure. A 6-mm Bergstrom biopsy needle was inserted through the skin and fascia incision into the muscle, and muscle tissue samples were obtained by manual suctioning and cutting with a coaxial blade. The biopsy specimen then was cut into small sections, which were snap frozen in liquid nitrogen and subsequently stored at -80°C until used for analyses.

### Physical Activity Assessment

Physical activity participation was determined using an interview-administered standardized questionnaire originally developed for the health, aging, and body composition study (Brach et al., 2004) and modeled after the leisure-time physical activity questionnaire (Taylor et al., 1978). Levels of moderate to vigorous physical activity was estimated from responses to questions concerning brisk walking (“walking at a fast pace where it may be difficult for you to speak normally, sometimes called power walking”), weight or circuit training activities and vigorous exercise activities (“like bicycling, swimming, running, aerobics, basketball, soccer, rowing, racquet sports, stair-stepping, elliptical or cross-country ski machine or exercycle”). Participation data for up to four vigorous activities was collected. For each type of activity, participants were asked whether they performed the activity in the past 2 weeks and, if so, the frequency performed, and average amount of time spent for each activity. Total participation time in moderate to vigorous physical activity per week was calculated by multiplying frequency by amount of time performed for each activity, summing all of the activities then dividing by two to derive minutes of moderate to vigorous physical activity per week. For analyses, the following categories were used: <30 min per week of high intensity physical activity was considered “not active” and coded as 0; high intensity physical activity ≥30 and <75 min was considered “moderately active” and coded as 1, high intensity physical activity ≥75 and <150 min was considered “active” and coded as 2, and high intensity physical activity ≥150 min was considered to “highly active” and coded as 3. The resulting ordinal variable from 0 to 3 was used in the analysis. Participation in casual walking was determined in a similar manner. One-way ANOVA, non-parametric, and chi-square tests (continuous and categorical variables) were used to test for sample differences between physical activity groups and age groups.

### Sample Preparation for LC-MS Analysis

Muscle samples were snap frozen in liquid nitrogen immediately after collection and stored at -80°C. On average, 8 mg of muscle tissue was pulverized in liquid nitrogen and lysed [8 M urea, 2 M thiourea, 4% CHAPS, 1% Triton X-100, 50 mM Tris (Sigma), pH 8.5] and sonicated. Protein concentration was determined using a 2-D Quant Kit (GE Healthcare Life Sciences). Detergents and lipids were removed using a methanol/chloroform extraction protocol (Bligh and Dyer, 1959). Proteins were resuspended in urea buffer [8 M urea, 2 M thiourea, 150 mM NaCl (Sigma)], reduced (50 mM DTT), and alkylated (100 mM iodoacetamide), then diluted 12 times with 50 mM ammonium bicarbonate, and digested {18 h at 36°C using a trypsin/LysC mixture [Promega, 1:50 (w/w)]}. Peptides were desalted, speed vacuum dried, and stored at -80°C. Tandem mass tag (TMT) labeling was performed according to the manufacturer’s instructions (TMT6plex, Thermo Fisher, Cat# 90066). Donor IDs were blinded and randomized to prevent TMT bias. Each TMT set included one donor from each of the 5 age groups and one reference sample. Each sample was spiked with 200 fM of bacterial beta-galactosidase digest (SCIEX) prior to TMT labeling. Labeled peptides were combined and fractionated.

### High-pH RPLC Fractionation and Concatenation Strategy

High-pH RPLC fractionation was performed as described previously (Wang et al., 2011). Skeletal muscle tissues samples were separated using an organic gradient (5–50% B, 100 min) into 99 fractions (1 min each) and merged into 33 master fractions (fraction 1, 34, 67 = master fraction 1, fraction 2, 35, 68 = master fraction 2, and so on). Combined fractions were speed vacuum dried, desalted, and stored at -80°C until final LC-MS/MS analysis.

### Nano LC-MS/MS Analyses

Skeletal muscle samples were analyzed using an UltiMate 3000 Nano LC Systems coupled to a Q Exactive HF mass spectrometer (Thermo Scientific, San Jose, CA, United States). Each fraction was separated by reverse phase on a 35-cm capillary column (3-μm C18, Hamilton, HxSil cat 79139) with 150 μm ID at 650 nL/min (5–30% B, 205 min). Tandem mass spectra were obtained using a Q Exactive HF mass spectrometer with a heated capillary temperature +280°C and spray voltage set to 2.5 kV. Full MS1 spectra were acquired from 300 to 1500 m/z at 120,000 resolution and 50-ms maximum accumulation time with automatic gain control set to 3 × 106. Dd-MS2 spectra were acquired using a dynamic m/z range with a fixed first mass of 100 m/z. MS/MS spectra were resolved to 30,000 with 155-ms of maximum accumulation time and the automatic gain control target set to 2 × 105. The 12 most abundant ions were selected for fragmentation using 30% normalized high collision energy. A dynamic exclusion time of 40 s was used to discriminate against the previously analyzed ions.

### Proteomics Informatics

Raw data from each sample fraction were converted to mascot generic format (.mgf) using MSConvert (ProteoWizard 3.0.6002), producing a list of MS ions with retention times and MS/MS spectra. This list of ions was searched with Mascot 2.4.1 and X!Tandem CYCLONE (2010.12.01.1) using SwissProt Human sequences from the Uniprot database (Version Year 2015, 20,200 sequences, appended with 115 contaminants). The search engine uses each protein sequence from the database to produce every possible peptide from it according to the following search parameters: TMT6plex lysine and n-terminus as fixed modifications and variable modifications of carbamidomethyl cysteine, deamidation of asparagine and glutamate, and carbamylation of lysine, the N-terminus, and oxidized methionine. These modifications were frequently set as variable modifications because they are often generated by sample preparation. A peptide mass tolerance of 20 ppm and 0.08 Da were allowed, according to the known mass accuracy of the instrument, for precursor and fragment ions, as well as two missed cleavages. The TMT channels’ isotopic purity was corrected according to the TMT Kit instructions.

Results from the Mascot and X!Tandem search engines were analyzed by Scaffold Q+ 4.4.6 (Proteome Software, Inc), and the peptide and protein probabilities were calculated by PeptideProphet and the ProteinProphet probability model (Keller et al., 2002; Nesvizhskii et al., 2003). The data were filtered at thresholds of 0.1% peptide false discovery rate (FDR), 1% protein FDR, and requiring a minimum of 1 unique peptide for protein identification. The experiments in this study compared proteins across multiple samples, identifying quantifiable peptides as only those detected across all samples (*n* = 58), reducing the probability of false peptide discovery. Finally, proteins were included for analysis even when identified from a single specific peptide, but only if the identification was confirmed by more than one search engine. As for single peptide quantification, the spectrum-to-spectrum variability was no higher between spectra from the same peptide than between spectra from different peptides from the same protein, reducing the likelihood of any differential “bias” in reporter ions from peptide to peptide. More importantly, TMT was taken as a relative, not absolute, quantification. Even if there were such a bias, it would be the same across samples and, thus, the relative quantification would not be affected. Spectral quantitative values were extracted from Scaffold and decoy spectra, while contaminant spectra and peptide spectra shared between more than one protein were removed. Typically, spectra are shared between proteins if the two proteins share most of their sequence, usually for protein isoforms. Spectra were retained for further analyses if they were exclusive to only one protein and were identified in all 6 channels across each TMT set. The log2 transformed spectral abundance was normalized by median subtraction from all reporter ion intensity spectra belonging to a protein across all channels. Relative protein abundance was estimated by the median of all peptides for a protein combined together. Protein sample loading effects from sample preparations were corrected by median polishing, i.e., subtracting the channel median from the relative abundance estimate across all channels to have a zero median (Herbrich et al., 2013; Kammers et al., 2015).

Linear mixed regression models were used to examine the effect of physical activity (considered as an ordinal variable) on each protein after adjusting for age, race, BMI, the ratio between type I (MYH7) and type II myosin fibers (MYH1, MYH2, and MYH4) in order to account for different fiber type composition, and TMT mass spectrometry experiments. The ratio of type I and type II fiber was estimated from the log2 normalized protein intensities of MYH7, MYH1, MYH2, and MYH4. The expression of these fiber types was calculated for each participant and the ratio of the fiber types was estimated as the ratio of MYH7 over the sum of MYH1, MTH2, and MYH4 protein intensity. The size of the beta coefficient for physical activity (as an ordinal variable) was used to quantify the effect of physical activity on each protein independent of covariates, and the significance of the association from the regression model was determined with *p*-values derived from lmerTest. Any protein *p* < 0.05 was considered to be significant and reported in the main text as a protein associated with physical activity. Multiple testing correction of the *p*-values were performed using the Benjamini-Hochberg method in R and reported in the supplement. The regression model was performed using R 3.3.4 (R Core Team, 2016) with the lme4 v1.1. library. Proteins with a negative beta coefficient were considered to be negatively associated with physical activity and the proteins with positive beta coefficient as positively associated with physical activity. Protein annotations were performed using GeneOntology, Uniprot keyword, and manual curation. Heat maps and hierarchical cluster analysis were performed using the non-linear minimization package in R (Gaujoux and Seoighe, 2010). GraphPad PRISM 6.07 and R packages were used for statistical analysis and generation of figures. The mass spectrometry proteomics data have been deposited to the ProteomeXchange Consortium via the PRIDE partner repository with the dataset identifier PXD011967 (Vizcaino et al., 2016).

## Results and Discussion

### Study Participation and Proteome Isolation From Muscle Biopsies

We sought to determine proteomic differences in the muscle tissues of 20–87 year-old subjects according to physical activity status, while also considering age and sex as covariates. Supplementary Table S1 details the characteristics of study participants by age group, while Table 1 contains the distribution of the self-reported frequency of major physical activity tasks according to physical activity groups. Not active participants tended to be younger, but age differences between groups were not statistically significant. On average, participants in each of the four groups were slightly overweight, but BMI did not differ between the physical activity groups. Due to the inclusion criteria, all participants were free of major diseases.

**Table 1:** Baseline characteristics of the GESTALT participants included in this study.

Physical activity group	Not active (0)	Moderately active (1)	Active (2)	Highly active (3)	*p*-value
		
	(*n* = 11)	(*n* = 8)	(*n* = 14)	(*n* = 25)	
Gender	*M*7, *F*4	*M*6, *F*2	*M*7, *F*7	*M16*, *F*9	0.686
Age, years	50.8 ± 21.6	58.3 ± 25	59.7 ± 18.8	52 ± 19.1	0.596
Race	6*C*, 4*AA*, 1*A*	4*C*, 3*AA*, 1*A*	11*C*, 3*AA*	20*C*, 4*AA*, 1*A*	0.51
BMI, kg/m^2^	25.7 ± 2.5	26.6 ± 3.3	25.1 ± 3.1	26.4 ± 3	0.558
^†^Weight/circuit training	0 ± 0	31 ± 11	77 ± 38	153 ± 194	0.1274
^†^Vigorous exercise	1 ± 4	4 ± 10	31 ± 42	171 ± 145	<0.0001
^†^Brisk walking	0 ± 0	29 ± 21	89 ± 117	140 ± 157	0.001
^†^Casual walking	121 ± 77	78 ± 75	254 ± 178	249 ± 227	0.051


*Gender, the number of participants is reported on top of each column; *M*, male; *F*, female. Age expressed in years as mean ± standard deviation. Race: *C*, Caucasian; *AA*, African American; and *A*, Asian. Body Mass Index (BMI) expressed as mean ± SD. ^†^Physical activity expressed in minutes/week as mean ± SD. The sample difference between physical activity groups were calculated by One-way ANOVA, non-parametric, and chi-square tests (continuous and categorical variables).*

### Identification of Physical Activity-Associated Proteins

**FIGURE 1:**
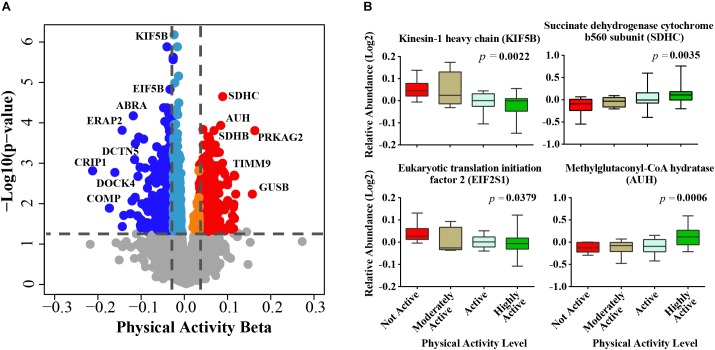
Physical activity associated proteins in human skeletal muscle. **(A)** Proteins associated with physical activity. Level of significance of each protein was assessed by lmerTest from the mixed model regression results and the protein was reported as significant if the *p* < 0.05. Red circles are proteins overrepresented with higher physical activity and blue circles are proteins underrepresented with higher physical activity. The proteins that were least affected by physical activity are shown as gray, light blue, and orange. **(B)** The average raw data protein levels are shown for most significantly overrepresented or underrepresented with higher physical activity, according to self-reported physical activity level. The left axis represents the log2 relative abundance and the *x*-axis the level of physical activity.

After adjusting for confounders, of the ∼4,300 proteins identified from the TMT experiments (Supplementary Table S2) 1,019 proteins were significantly associated with physical activity (*p* < 0.05) (Supplementary Table S3). The beta coefficients for proteins associated with physical activity (indicating log2 fold expression across physical activity levels) ranged from -0.22 (gene CRIP1) to 0.15 (gene PRKAG2), where negative and positive beta coefficients indicated proteins which log2 abundances were lower or higher, respectively, with higher physical activity. Of the 1,019 physical activity-related proteins, 324 and 695 proteins were upregulated and downregulated, respectively (Figure 1A). The top proteins that were positively associated with physical activity were mitochondrial proteins involved in energy metabolism or fatty acid oxidation, such as succinate dehydrogenase cytochrome b560 subunit (SDHC), methylglutaconyl-CoA hydratase (AUH), succinate dehydrogenase (SDHB), and isobutyryl-CoA dehydrogenase (ACAD8). SDHC is a complex II protein that declines with age (Melov et al., 2007) and has been reported to increase with physical activity (Figure 1B). Isobutyryl-CoA dehydrogenase, which was increased with physical activity in this study, has been reported to be downregulated in obesity in a twin study (Pietilainen et al., 2008).

The top significant protein that was negatively associated with physical activity was kinesin-1 heavy chain (KIF5B, beta = -0.0281, *p* = 6.61E-07) (Figure 1B), which is implicated in the transport of membrane organelles and other cellular cargoes along microtubules and has been associated with axonal outgrowth, neuroplasticity, and neurogenesis. Kinesin-1 also affects mitochondrial morphology by moving mitochondria along microtubule tracks away from the mitochondrial reticulum, leading to mitochondrial fragmentation (Iqbal and Hood, 2014). Wang et al. (2013) used a mouse model with a muscle-conditional knock-out for kinesin-1 to reveal severe muscle dystrophy, suggesting that KIF5b plays significant roles in myofibrillogenesis and in myotendinous junction stability. In linkage family studies, single nucleotide polymorphisms in KIF5B positively regulated the cardiac response to acute training (Argyropoulos et al., 2009). In addition, KIF5B has been recently identified as a novel gene associated with amyotrophic lateral sclerosis (Nicolas et al., 2018). Although this discovery may appear to be counterintuitive, it is consistent with the increased KIF5B gene expression observed in a rat model of sciatic nerve ligation (Kazemi et al., 2016). Thus, our findings suggest that the skeletal muscle from individuals who are physically active have low rates of fiber denervation, which no longer requires KIF5B upregulation to drive reinnervation (Mosole et al., 2014).

Cysteine-rich protein 1 (CRIP1, beta = -0.217, *p* = 0.0016) was also strongly underrepresented in muscle from persons who were highly physically active. CRIP1 is a zinc-finger protein involved in the cytokine-mediated immune response (Lanningham-Foster et al., 2002) and in cell proliferation (Louis et al., 1997). In a previous study of gene expression in blood peripheral cells, CRIP1 was one of the genes most highly upregulated with aging, possibly as a part of the pro-inflammatory state of aging that tends to be attenuated by physical activity (Nakamura et al., 2012).

### The Mitochondrial Proteome Is Upregulated With Higher Physical Activity Independent of Age

**FIGURE 2:**
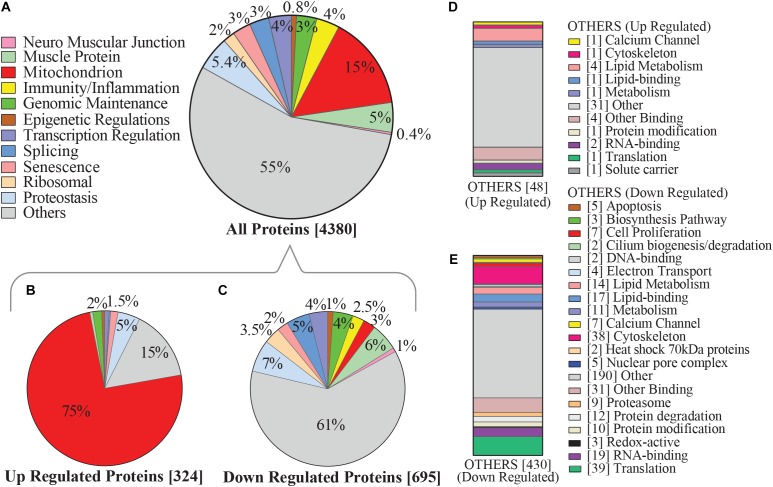
Categorization of physical activity associated hallmarks of aging proteins in human skeletal muscle. **(A**) Categories of proteins which were detected by mass spectrometry are shown, focusing on pathways that emerge from aggregated clusters of proteins and have been associated with aging and/or physical activity. Level of significance of each protein was assessed by lmerTest from the mixed model regression results and the protein was reported as significant if the *p* < 0.05. **(B)** Proteins most significantly upregulated with higher physical activity, of which 75% were mitochondrial proteins. **(C)** 695 proteins were significantly underrepresented with higher physical activity, including those connected with proteostasis, splicing, and immunity/inflammation. **(D)** Subcategorization of the 48 upregulated “others” category proteins from **B**, which were not considered in detail in this study. **(E)** Subcategorization of the downregulated “others” category proteins from **C** not considered in detail in this study, including protein classes associated with cell proliferation, cytoskeleton, lipid metabolism, and translation. Square bracket on left shows number of proteins associated in each category.

In this study, mitochondrial proteins represented roughly 15% of all proteins detected from muscle biopsies. Roughly 40% of all mitochondrial proteins were differentially regulated based on level of self-reported physical activity (Figure 2A) and as many as 75% of these proteins were significantly overrepresented with higher physical activity (Figure 2B,D). This is consistent with established effects of exercise on mitochondrial biogenesis, suggesting that routine, daily physical activity is sufficient to improve mitochondrial muscle content, and may counteract diminishing oxidative capacity in skeletal muscle that has been previously described with normal aging and is associated with mobility loss (Lanza et al., 2008; Schild et al., 2015; Choi et al., 2016; Deschenes and Chabot, 2017; Robinson et al., 2017). Conversely, downregulated proteins were evenly distributed throughout various functional categories (Figure 2C,E).

**FIGURE 3:**
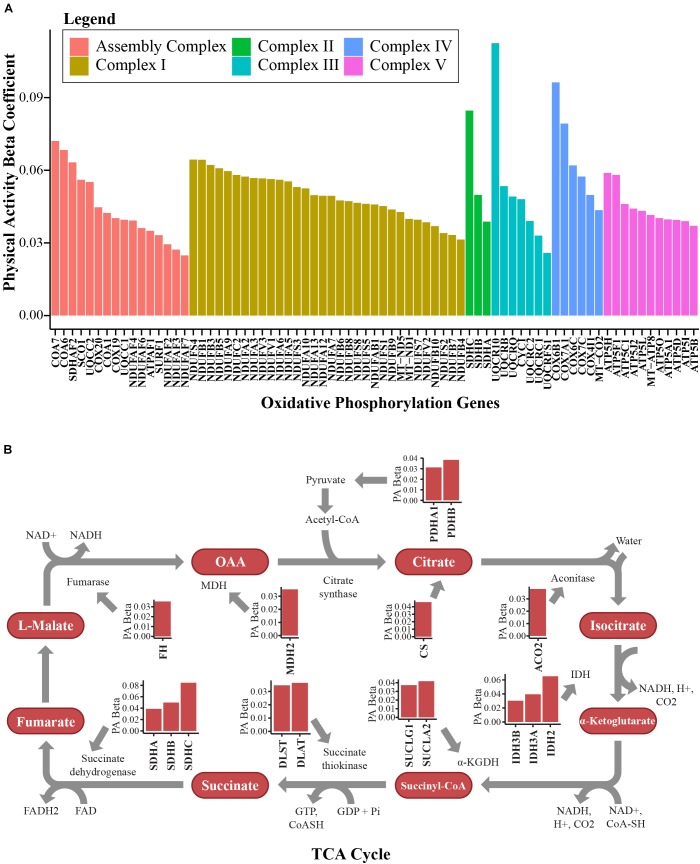
Bioenergetics pathways upregulated with higher physical activity in human skeletal muscle. **(A)** Proteins associated with oxidative phosphorylation. Proteins from complex I, complex II, complex III, complex IV, and complex V in the electron transport chain and assembly complex proteins were overrepresented with higher physical activity. The *y*-axis shows the log2 abundance change of the protein associated with one higher point in the physical activity score (0–3) and the *x*-axis shows the gene name associated with each protein. Different complexes are color coded. **(B)** Many TCA cycle proteins were overrepresented with higher physical activity. All significantly quantified proteins are shown as bar plots. Level of significance of each protein was assessed by lmerTest from the mixed model regression results and the protein was reported as significant if the *p* < 0.05.

The majority of the upregulated mitochondrial proteins associated with higher physical activity were from the mitochondrial inner membrane (36.5%). Both structural proteins and enzymes in the mitochondrial inner membrane play fundamental roles in transducing energy through oxidative phosphorylation to produce ATP (Saraste, 1999; Huttemann et al., 2007). In response to high activity, proteins from the electron-transportation chain (ETC) were upregulated. The top upregulated proteins across the 5 complexes were NADH-dehydrogenase from complex I, SDHB from complex II, cytochrome *c* from complex III, cytochrome *c* oxidase from complex IV, and ATP synthase for complex V (Figure 3A). Beyond the ETC protein components, assembly factor proteins for the mitochondrial complex subunits were upregulated, especially those involved in complex I biogenesis (Figure 3A). Interestingly, the mitochondrial transcription factor A (TFAM, beta = 0.045, *p* = 0.0026) was also upregulated (beta = 0.045, *p* = 0.0026). This is consistent with the notion that exercise stimulates the activity of peroxisome proliferator-activated receptor gamma co-activator-1 alpha (PGC-1α), which in turn stimulates the transcription of TFAM (Perry and Hawley, 2018). TFAM binds to specific mtDNA sites and promotes the transcription and replication of mtDNA, which ultimately results in mitochondrial biogenesis (Bengtsson et al., 2001).

Mitochondrial carriers are essential for supplying the substrate that is essential for proper ETC function. From this study, 10 mitochondrial solute carrier proteins were overrepresented with physical activity, namely SLC16A1, SLC25A10, MPC2, SLC25A4, SLC25A3, SLC25A20, SLC25A19, SLC25A11, SLC25A12, and SLC25A5. SLC25A4 is particularly interesting, as it is the main energy shuttle that imports ADP from the cytosol and exports ATP produced in the mitochondrial matrix by ATP synthase. Two other important proteins overrepresented with physical activity should also be noted. The first, creatine kinase S-type (CKMT2, beta = 0.054, *p* = 0.0297), has been implicated in transporting high energy phosphate produced by oxidative phosphorylation to the cytosol in muscle cells in the form of phosphocreatine. The second is adenylate kinase 2 (AK2, beta = 0.034, *p* = 0.0196), which catalyzes the formation of ADP from ATP and AMP, a main sensor of cellular energy homeostasis and an essential process in adenine nucleotide metabolism. Energy production in mitochondria through the ETC requires adequate function of the TCA cycle. Fourteen TCA cycle proteins were significantly (*p* < 0.05) upregulated in skeletal muscle with physical activity (Figure 3B).

Human sirtuins (SIRTs) are a heterogeneous class of proteins that have ADP-ribosyltransferase, deacetylase or deacylase activity, and these proteins have been implicated in aging, metabolic regulation, mitochondrial biogenesis, and inflammation, leading to them being considered promising therapeutic targets (Bonkowski and Sinclair, 2016). SIRTs require NAD+ and are thereby considered to be sensors of metabolic and energy status (Chang and Guarente, 2014). In this study, SIRT3 (beta = 0.05, *p* = 0.0007) and SIRT5 (beta = 0.037, *p* = 0.01) – both of which are localized to the mitochondria and regulate energy metabolism, playing an important role in regulating mitochondrial biogenesis – were overrepresented in persons with higher physical activity. SIRT3 is the most potent deacetylase among known sirtuins and previous studies have indicated that SIRT3 concentration declines with aging and increases with physical activity (Lanza et al., 2008; Munoz et al., 2018). Recent data revealed that SIRT5 modulates a TCA cycle intermediate via alternate lysine modifications - including succinylation and malonylation – and modulates enzymes that control fatty acid beta oxidation and the TCA cycle in skeletal muscle fibers (Bringman-Rodenbarger et al., 2018). Overall, our findings demonstrate that high physical activity has a strong effect on mitochondrial proteins, including ETC proteins and TCA cycle regulation proteins, in addition to proteins that contribute to maintaining ETC integrity and function.

### Physical Activity Is Associated With Lower Representation of Immune Proteins and Inflammatory Mediators

Intense exercise has a strong effect on immune function; an acute bout of exercise is associated with proinflammatory cytokine production and immunodepression during recovery from fatigue (Peake et al., 2017; Windsor et al., 2018). In contrast, individuals who exercise regularly tend to have enhanced immune function and less evidence of a proinflammatory state (Sellami et al., 2018). In this study, we found that several innate immune proteins linked to macrophage function were negatively associated with physical activity, including macrophage capping protein (CAPG), WD repeat and FYVE domain containing 1 protein (WDFY1), Chitinase domain-containing protein 1 (CHID1), myosin 18A (MYO18A), Inositol polyphosphate phosphatase-like 1 (INPPL1), and Poly(rC)-binding protein 2 (PCBP2) (Table 2). Proteins that belong to the complement system, another component of innate immunity, were also downregulated in muscle from participants who reported higher physical activity. These included complement factors C1RL and CFD, as well as complement c1Q binding protein (Table 2). Finally, high-mobility group box-1 protein (HMGB1), a redox sensitive protein which promotes leukocyte recruitment to the muscle and activates TLR4 via downstream chemokine and cytokine release, was also underrepresented with higher physical activity (Vezzoli et al., 2010). Overall, regular physical activity in daily life appears to be associated with lower activation of innate immunity response. However, it remains unclear whether the downregulation of innate immunity is direct or mediated by the accumulation of intramuscular adipose tissue in skeletal muscle, which occurs more frequently in sedentary individuals and is notoriously pro-inflammatory (Ferrucci and Fabbri, 2018).

**Table 2:** Immune related proteins and physical activity.

Gene name	Uniprot ID	Location	Protein description	Physical activity beta	Physical activity *p*-value
ERAP2	ERAP2_HUMAN	Endoplasmic reticulum	Endoplasmic reticulum aminopeptidase 2	–0.1486	0.0002
IGHA2	IGHA2_HUMAN	Cell membrane	Immunoglobulin heavy constant alpha 2	–0.1291	0.0184
IGKC	IGKC_HUMAN	Cell membrane	Immunoglobulin kappa constant chain C region TI	–0.1063	0.0360
C1RL	C1RL_HUMAN	Other	Complement C1r subcomponent-like protein	–0.0661	0.0454
CAPG	CAPG_HUMAN	Nucleus	Macrophage-capping protein	–0.0594	0.0422
CFD	CFAD_HUMAN	Other	Complement factor D	–0.0591	0.0184
WDFY1	WDFY1_HUMAN	Nucleus	WD repeat and FYVE domain-containing protein 1	–0.0397	0.0135
OTUD7B	OTU7B_HUMAN	Nucleus	OTU domain-containing protein 7B	–0.0350	0.0033
CHID1	CHID1_HUMAN	Lysosome	Chitinase domain-containing protein 1	–0.0329	0.0064
DBNL	DBNL_HUMAN	Cell membrane	Drebrin-like protein	–0.0324	0.0378
UBE2V1	UB2V1_HUMAN	Nucleus	Ubiquitin-conjugating enzyme E2 variant 1	–0.0311	0.0148
OTUB1	OTUB1_HUMAN	Nucleus	Ubiquitin thioesterase OTUB1	–0.0294	0.0007
MYO18A	MY18A_HUMAN	Endoplasmic reticulum	Unconventional myosin-XVIIIa	–0.0285	0.0083
INPPL1	SHIP2_HUMAN	Membrane	Phosphatidylinositol 3,4,5-trisphosphate 5-phosphatase 2	–0.0206	0.0042
ABCC9	ABCC9_HUMAN	Membrane	ATP-binding cassette sub-family C member 9	–0.0202	0.0470
EIF2AK2	E2AK2_HUMAN	Nucleus	Interferon-induced, double-stranded RNA-activated protein kinase	–0.0188	0.0115
PCBP2	PCBP2_HUMAN	Nucleus	Poly(rC)-binding protein 2	–0.0148	0.0297
NLRX1	NLRX1_HUMAN	Mitochondrion outer membrane	NLR family member X1	0.0183	0.0343
C1QBP	C1QBP_HUMAN	Mitochondrion matrix	Complement component 1 Q subcomponent-binding protein	0.0362	0.0066


*Immune related proteins were significantly underrepresented in skeletal muscle of participants who reported higher levels of physical activity. Level of significance of each protein was assessed by lmerTest from the mixed model regression results and the protein was reported as significant if the *p* < 0.05. Of note, all immune proteins were negatively correlated with physical activity (negative beta), except for NLRX1, and C1QBP.*

Proteins indicative of adaptive immunity were also underrepresented with higher physical activity. These included regulators of antigen processing endoplasmic reticulum aminopeptidase 2 (ERAP2) (de Castro and Stratikos, 2018), and T-cell activation drebrin-like protein (DBNL) (Le Bras et al., 2004). Other proteins that modulate NFκB signaling were also downregulated, including ubiquitin conjugating enzyme E2 V1 (UBE2V1), which activates NFκB (Syed et al., 2006; Flowers et al., 2018), and OTU deubiquitinase 7B (OTUD7B), a clock-regulated protein that downregulates the non-canonical NF-κB pathway (Hou et al., 2017; 48: 939–950). Lower levels of immunoglobulin heavy constant alpha 2 (IGHA2) and immunoglobulin kappa constant (IGKC) in muscle of individuals with higher physical activity indicated reduced recruitment of B lymphocytes (Table 2). Unsurprisingly, unlike all other immune proteins, NLR family member X1 (NLRX1), a protein that regulates mitochondrial antivirus response that is located on the outer mitochondrial membrane, was positively associated with physical activity (Jaworska et al., 2014).

High intensity training may cause mechanical damage to skeletal muscle, followed by an inflammatory response and increased levels of proteins connected with apoptosis and necrosis (Podhorska-Okolow et al., 1998). However, this was not observed with participants in this study, even those who were engaged in high intensity training, as proteins responsible for inflammatory response and apoptosis were downregulated, likely due to relatively little acute damage. For example, higher physical activity was associated with decreased expression of WD repeat domain 92 (WDR92, beta = -0.027, *p* = 0.0485), B cell receptor associated protein 31 (BCAP31, beta = -0.029, *p* = 0.001), and phosducin-like 3 (PDCL3, beta = -0.037, *p* = 0.00008) – proteins which exert pro-apoptotic activity by interacting with different caspases. Similarly, B-cell lymphoma 2 protein – a strong pro-apoptotic protein that opens a mitochondrial voltage-dependent anion channel that causes loss of membrane potential and cytochrome *c* release – was also downregulated. However, proteins described as anti-apoptotic, such as myeloid derived growth factor (MYDGF, beta = -0.047, *p* = 0.006) and major facilitator superfamily domain containing 10 (MFSD10, beta = -0.089, *p* = 0.010), were also lower with higher physical activity, consistent with the apoptosis mechanism being balanced in physically active individuals (Phaneuf and Leeuwenburgh, 2001).

**FIGURE 4:**
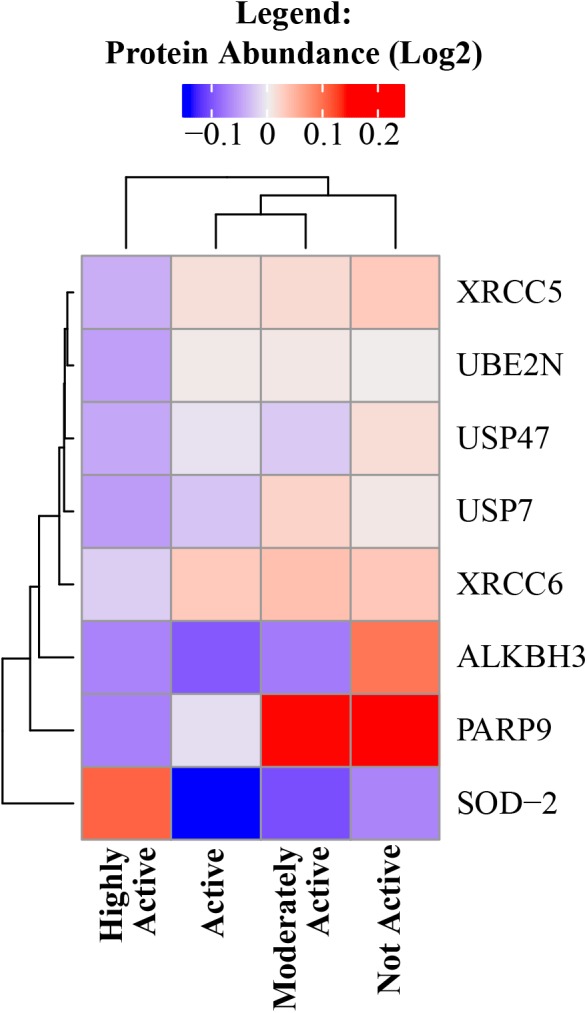
DNA damage and DNA repair proteins decreased in abundance with higher physical activity. All significant DNA damage and DNA repair proteins were negatively correlated with physical activity except SOD-2. The level of significance of each DNA damage protein was assessed by lmerTest from the mixed model regression results and the protein was reported as significant if the *p* < 0.05. Heat map shows four physical activity levels (*x* axis) and the average abundance of significant DNA damage and DNA repair proteins correlated with physical activity levels (*y* axis). Dark blue suggests lower relative log2 abundance of the protein and red suggests higher log2 relative protein abundance. The range of log2 abundance of the protein is from –0.1 to 0.2 (legend on top). The similarity between genes and physical activity groups are represented by hierarchical clustering on *y*- and *x*-axis, respectively.

Exercise can cause an acute increase in energetic metabolism that unavoidably results in greater production of reactive oxygen species (ROS) by mitochondria, which can cause oxidative damage to DNA, proteins, and other macromolecules. However, as an individual continues to exercise, multiple antioxidant mechanisms are upregulated to buffer the damaging effects of ROS (Gomez-Cabrera et al., 2008; Hitomi et al., 2008; He et al., 2016). Indeed, superoxide dismutase (SOD) acts as the first line of defense in skeletal muscle against oxidative stress and SOD-2 is significantly overrepresented with higher physical activity (beta = 0.04, *p* = 0.005). In accordance with this view, a number of DNA damage and DNA repair proteins decreased in abundance with higher physical activity – such as poly(ADP-ribose) polymerase family member 9, alkylated DNA repair protein AlkB homolog 3, X-ray repair cross-complementing 5 and 6 (Lu et al., 2018), ubiquitin conjugating enzyme E2 N, and replisome-enriched small ubiquitin-like modifiers deubiquitinase – which are essential for DNA replication (Figure 4).

Senescent cells accumulate progressively with aging in tissues and organs (He and Sharpless, 2017). Current research is focused on determining if terminally differentiated, non-replicating cells – such as myocytes – may develop a specific form of senescence, while the impact of age and physical activity on senescence phenotypes in muscle is also under investigation. In this study, several proteins that are annotated as senescence-associated were underrepresented in participants who reported higher levels of physical activity (e.g., MAP2K3 and EIF3A), with the exception of mitochondria-localized senescent proteins, which were instead overrepresented (Supplementary Table S4). The role of these proteins in the changes that occur in intermyocellular adipocytes, infiltrated immune cells, or myocytes – or rather in satellite cells, which is unlikely given the small volume of these cells – is unclear and should be addressed by future studies.

### Physical Activity Affects the Spliceosome

**FIGURE 5:**
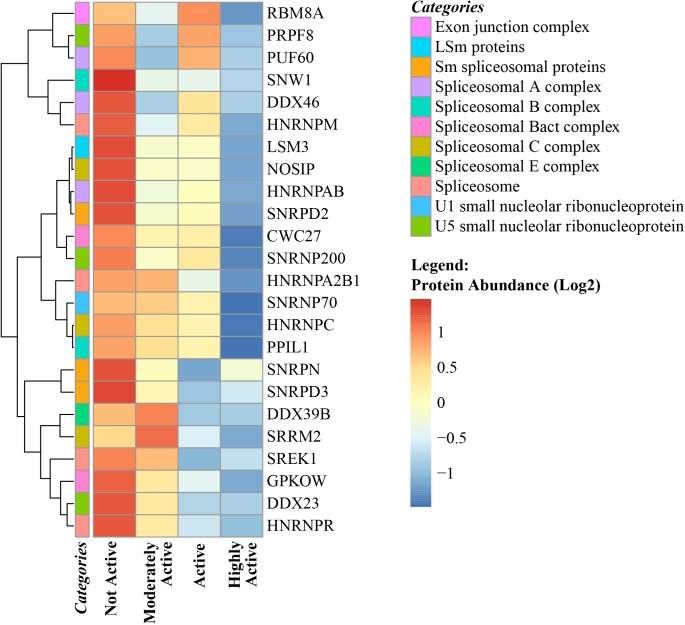
Spliceosome complex proteins in skeletal muscle and physical activity. All significant spliceosome complex proteins were negatively correlated with physical activity. The level of significance of each spliceosome protein was assessed by lmerTest from the mixed model regression results and the protein was reported as significant if the *p* < 0.05. The heat map correlates four physical activity levels (*x*-axis) with the average abundance of spliceosomes genes correlated with physical activity levels (*y*-axis). Dark blue suggests negative correlation and red suggests positive correlation. The normalized range of the protein abundance for physical activity correlation is shown from red to blue (legend on bottom right). The color-coded “categories” column of the heatmap shows the spliceosome complex category annotated for each gene and the spliceosome category annotation label legend on top right shows the spliceosome major complex categories. Similarity between the spliceosome genes are shown by hierarchical clustering and represented with the dendrogram on the left *y*-axis.

The spliceosome is organized into 6 different well-recognized protein complexes (Matera and Wang, 2014) that process pre-mRNA transcripts and cause different combinations of exons (and sometime introns) to produce a variety of splicing protein variants from the same gene. Alternative splicing in skeletal muscle regulates myogenesis, muscle contraction, and calcium handling in myofibers. There are ∼70 genes that undergo both transcriptional and splicing regulation during myogenesis (Trapnell et al., 2010) and 95 alternative splicing events that undergo robust and conserved splicing changes during the course of myogenic differentiation (Bland et al., 2010). The number of genes that are differentially spliced and the number of splicing errors that produce non-functional proteins tend to increase with age (Rodriguez et al., 2016; Saudemont et al., 2017). Previous studies have proposed that changes in alternative splicing with aging may be reflective of an adaptive strategy aimed at compensating for the accumulation of molecular damage (Latorre and Harries, 2017). In this study, ∼99 spliceosome proteins were quantified, of which 24 were significantly downregulated with physical activity (Figure 5). Speculatively, this may indicate that the requirements for compensation are reduced due to less accumulated damage. However, there is limited evidence connecting abundance of splicing machinery proteins and changes in the number and type of splicing variants (such as progerin) produced during transcription and translation related to senescence (Deschenes and Chabot, 2017). Interestingly, the abundance of the aging-related laminin-A splice variant progerin (beta = -0.023, *p* = 0.0063) was lower in highly active individuals along with other spliceosome proteins. Although a causal relationship between downregulation of splicing proteins and lower progerin levels (in addition to other splicing variants) cannot be established from our observational data, this hypothesis should be addressed in future studies. Nevertheless, the data from this study suggest that changes in the splicing machinery that occur with aging might be offset by regular physical activity, although the relevance to human health of this effect remains to be established.

### Physical Activity Affects Autophagy and Proteostasis

Muscle contraction during exercise increases intracellular calcium and causes mechanical and chemical stresses that trigger the unfolded protein response (Vainshtein et al., 2014). In addition, the accelerated pace of oxidative phosphorylation increases ROS production, damaging membrane lipids and proteins, while also potentially impairing organellar function, including mitochondria. This potential accumulation of damage is counteracted by the activation of macroautophagy and chaperone-mediated autophagy (Grumati et al., 2011; He et al., 2012). Contrary to previous observations, this study revealed that proteins involved in the activation/inhibition of autophagy – such as V-type proton ATPase 116 kDa subunit a isoform 1 (ATP6V0A1), apoptosis regulator BAX, biogenesis of lysosome-related organelles complex 1 subunit 1 (BLOC1S1), heat shock protein HSP 90-alpha (HSP90AA1), Ras-related protein Rab-7a (RAB7A), and heat shock cognate 71 kDa protein (HSPA8 ) – were underrepresented in muscle biopsies from participants who were highly active. In contrast, β-glucuronidase (GUSB), an important lysosomal enzyme, and 5′-AMP-activated protein kinase subunit gamma-2 (PRKAG2), a component of AMP kinase that is the main energy-sensor that responds to changes in the cellular AMP:ATP ratio and regulates the balance between ATP production and consumption, were greater with higher physical activity, suggesting a tightly monitored balance between energy production and utilization (Mounier et al., 2015). Overall, 62 proteins related to proteostasis were quantified and were significantly associated with physical activity; 47 were underrepresented with higher physical activity, including 6 chaperonins, 20 chaperones, including 3 heat shock proteins 70, 3 heat shock proteins 90 and 1 small heat shock protein (sHSP), 7 co-chaperones, 6 DNAJ (HSP40), and 8 other proteostasis proteins (Supplementary Table S5). Of the 62 noted proteostasis proteins, 15 proteins were overrepresented in the muscle from participants who reported higher levels of physical activity, including 7 chaperones (including an HSP70), 3 DNAJ (HSP40) proteins, and 5 other proteostasis proteins. DNAJB2 transcript has been previously reported to be upregulated in human skeletal muscle during recovery from exercise-induced damage (Mahoney et al., 2008). sHSPs play an important role in cytoskeleton protection and a bout of eccentric exercise induces rapid accumulation of sHSPs (HSPB1 and HSPB5) and later accumulation of HSP70 (Paulsen et al., 2007). Our study revealed that some specific chaperones and sHSPs were underrepresented, while others were overrepresented based on physical activity, which could due to differences between selective cell structures when reacting to regular physical activity that induces significant levels of damage accumulation. Further investigation is required to clarify this discrepancy.

### Summary of Conclusion

In this study, we used discovery proteomics to understand how daily physical activity may affect skeletal muscle biology. This approach is substantially different from previous proteomic studies that have characterized the effect of acute increased physical activity or intense training, which can be conceptualized as a dynamic response to stress that is aimed at reaching a different level of equilibrium (Margaritelis et al., 2018). In contrast, this study examined individuals who had not dramatically changed their level of physical activity over the last few months, in order to understand what adaptive changes are maintained in active muscle over a long period. It must be noted that, although study participants were 20–87 years of age, they were all extremely healthy according to very strict and objective inclusion criteria and all individuals were not medicated. The most evident result, namely that physical activity was associated with higher structural and functional mitochondrial proteins, was not unexpected. However, it is important to note that none of the participants had performed an acute bout of intense physical activity directly before the muscle biopsy and, therefore, these findings demonstrate that maintaining a good level of regular physical activity may be sufficient to counteract the declining mitochondrial health that occurs in many aging individuals, which can result in negative metabolic and functional consequences (Choi et al., 2016; Zane et al., 2017). Interestingly, the expanded energy machinery was not associated with increased DNA damage due to oxidative stress, as evidenced by unchanged levels in DNA damage/repair proteins. Consistent with previous studies, there was evidence of enhanced antioxidant mechanisms, including overexpression of SOD and SOD-2 (de Sousa et al., 2017; Bouzid et al., 2018).

Interestingly, immune-related proteins were underrepresented in the muscle of participants who were physically active, including proteins related to both innate and adaptive immunity, which is also consistent with the literature and opposes the proinflammatory state that is typical of aging (de Sousa et al., 2017). For example, Duggal et al. examined the immune profiles of 125 middle-aged and older adults who had been active cyclers for much of their adult lives, comparing them to 75 age-matched sedentary controls, revealing that physical activity protects against many aspects of immunosenescence, including thymic involution, and circulating levels of inflammatory markers (Duggal et al., 2018).This is the first study to demonstrate that usual physical activity in daily life is associated with lower inflammation in the muscle of healthy individuals. Inflammation is associated with satellite cell dysfunction and impaired muscle regeneration and, thus, increased inflammation may be one of the mechanisms that leads to sarcopenia with aging (Perandini et al., 2018). Indeed, interventions aimed at reducing inflammation have been proposed for preventing age-associated sarcopenia, although currently there is no solid evidence regarding efficacy (Alturki et al., 2018).

Unexpectedly, this study revealed a strong association between higher physical activity and lower expression of spliceosome complex proteins. Previous studies have demonstrated that expression of dysregulated splicing factor is associated with aging in humans, although the direction of this association remains controversial (Lee et al., 2016). However, this is the first study to show that physical activity is associated with a lower representation of spliceosome in skeletal muscle. The rationale for changes in spliceosome and how these changes differ from those that occur in normal aging are unknown. Alternative splicing is one mechanism used by mammals to counteract the damage accumulation that occurs with stress and aging (Li et al., 2017). Thus, physical activity may prevent damage accumulation in muscle and simultaneously prevents the activation of this potential mechanism of resilience. In this study, changes in splicing machinery proteins were complemented with data on qualitative or quantitative differential representation of splicing variants and this is an important direction for future research.

This study has unique strengths that serve as an important addition to the literature. First, this study utilized a relatively large sample of individuals who were extremely healthy based on strict, highly standardized criteria. Further, we used a quantitative proteomics approach using isobaric tags, which allowed for the quantification of a large set of proteins. The findings described and discussed herein are robust because they are not based on changes in one single protein, but instead are based on detecting harmonic changes in a large set of proteins that cluster in the same pathways. However, this study does have limitations, especially in terms of population sample, and will need to be replicated in an independent sample before the findings can be generalized. Also, although we used strict, highly standardized criteria to define healthy status in our study population, there is no guarantee that younger people who meet such criteria will remain healthy through mid- and late-life, and therefore, the results from this study need to be confirmed longitudinally. Finally, this study was conducted using a very healthy population in order to increase homogeneity of the findings and to avoid potential confounding effects of disease and treatments. Thus, our findings may not apply to persons who are affected by disease or are chronically medicated.

In conclusion, our study provides evidence that high levels of physical activity counteract some of the effects of aging, namely the decline in mitochondrial volume (and function) and in pro-inflammatory status. We also revealed a robust change in the splicing machinery, the functional meaning of which should be addressed in future studies.

## Data Availability

The datasets generated for this study can be found in PRIDE, dataset identifier PXD011967.

## Author Contributions

LF, RS, ES, and CU-M designed the study and performed the analyses. LF, CC, and MG-F performed and collected the muscle biopsies. AL, CU-M, and RM generated the data. All authors analyzed the data, wrote the manuscript, and gave final approval for publication.

## Conflict of Interest Statement

The authors declare that the research was conducted in the absence of any commercial or financial relationships that could be construed as a potential conflict of interest.
