# Supplementary material for: Physical Activity Associated Proteomics of Skeletal Muscle: Being Physically Active in Daily Life May Protect Skeletal Muscle From Aging
Source: Front Physiol. 2019 Mar 26;10:312. doi: 10.3389/fphys.2019.00312 (PMC6443906; doi:10.3389/fphys.2019.00312)
Supplement: Supplementary file 1 [file Data_Sheet_1.PDF]

Age Group	20-34 (*n*=13)	35-49 (*n*=11)	50-64 (*n*=12)	65-79 (*n*=12)	80+ (*n*=10)	*P*-value
**Gender**	*M*8, *F*5	*M*7, *F*4	*M*7, *F*5	*M*8, *F*4	*M6*, *F4*	0.9949
**Age, year**	27.2 ± 3.3	41.3 ± 4.5	57.1 ± 4.7	70.3 ± 2.3	82.4 ± 2.4	<0.0001
**Race**	9*C*, 2*AA*, 2*A*	5*C*, 6*AA*	8*C*, 4*AA*	10*C*,1*AA*,1*A*	9*C*,1*AA*	0.0958
**BMI, kg/m^2**	25.9 ± 2.8	26.4 ± 2.6	26.6 ± 3.2	26.4 ± 2.4	25.2 ± 3.9	0.9533
**Weight/circuit training***	166 ± 188	301 ± 391	90 ± 45	62 ± 56	48 ± 10	0.3777
**Vigorous exercise***	151 ± 176	33 ± 53	30 ± 66	120 ± 131	135 ± 158	0.0857
**Brisk walking***	66 ± 95	79 ± 86	130 ± 205	77 ± 120	123 ± 165	0.8957
**Casual walking***	158 ± 128	195 ± 170	310 ± 284	172 ± 165	209 ± 156	0.4825
**†Physical Activity**	1.8 ± 1.4	1.8 ± 1.3	2 ± 1.1	2.3 ± 1	1.5 ± 1.1	0.5145

Table S1) Baseline characteristics of the GESTALT skeletal muscle age group donors. The participants are classified into 5 different age groups. Gender: The number of donors is represented in numeric, *M* is Male, *F* is Female. Age in years: Average age across group and standard deviation (SD ±) for each age group. Race: number of donors is shown in left and race is shown in italics, *C* is Caucasian, *AA* is African American, and *A* is Asian. Body Mass Index (BMI): average BMI across the age group and SD (±) for each group is shown.

*Activity expressed in minutes/week as mean ± SD

†Physical activity is calculated from the sum of weight circuit, vigorous exercise, brisk walking and casual walking as high intensity exercise min/wk (highxmin). This is further categorized into 0,1, 2, and 3 ; 0 = highxmin < 30, 1 = 30 ≤ highxmin < 75, 2 = 75 ≤ highxmin < 150, 3 = 150 ≤ highxmin.
